# Experiences with VHA care: a qualitative study of U.S. women veterans with self-reported trauma histories

**DOI:** 10.1186/s12905-017-0395-x

**Published:** 2017-05-30

**Authors:** Shannon M. Kehle-Forbes, Eileen M. Harwood, Michele R. Spoont, Nina A. Sayer, Heather Gerould, Maureen Murdoch

**Affiliations:** 1 National Center for PTSD Women’s Health Sciences Division at VA Boston Healthcare System, Boston, MA 02130 USA; 2 Center for Chronic Disease Outcomes Research, Minneapolis VA Healthcare System, Minneapolis, MN 55417 USA; 3 Department of Medicine, University of Minnesota Medical School, Minneapolis, MN 55455 USA; 4 Division of Epidemiology and Community Health, University of Minnesota School of Public Health, Minneapolis, MN 55455 USA; 5 Pacific Islands Division, National Center for PTSD, Honolulu, HI 96819 USA; 6 Department of Psychiatry, University of Minnesota Medical School, Minneapolis, MN 55454 USA; 7 Department of Psychology, College of Liberal Arts, University of Minnesota, Minneapolis, MN 55455 USA; 8 Section of General Internal Medicine, Minneapolis VA Healthcare System, Minneapolis, MN 55417 USA; 9 One Veterans Drive (152), Minneapolis, MN 55417 USA

**Keywords:** Women, Veterans, Qualitative research, Patient satisfaction, Posttraumatic stress disorder, Military sexual assault

## Abstract

**Background:**

Women veterans in the United States, particularly those with posttraumatic stress disorder (PTSD) or a history of military sexual assault, have unique health care needs, but their minority status in the US Veterans Health Administration (VHA) has led to documented healthcare disparities when compared to men. This study’s objective was to obtain a richer understanding of the challenges and successes encountered by women veterans with self-reported service-related trauma histories (particularly those with a history of military sexual assault and/or posttraumatic stress symptomology) receiving VHA care.

**Methods:**

Thirty-seven female Vietnam and post-Vietnam (1975–1998) era veterans were randomly selected from a cohort of PTSD disability benefit applicants to complete semi-structured interviews in 2011–2012. Grounded-theory informed procedures were used to identify interview themes; differences between veterans with and without a history of military sexual assault were examined through constant comparison.

**Results:**

At the time of the interviews, many women believed that VHA was falling short of meeting women veterans’ needs (e.g., lack of women-only mental health programming). Also common, but particularly among those with a military sexual assault history, was the perception that VHA’s environment was unwelcoming; being “surrounded by men” yielded emotions ranging from discomfort and mistrust to severe anxiety. A few veterans reported recent positive changes and offered additional suggestions for improvement.

**Conclusions:**

Findings suggest that while at the time of the interviews gains had been made in the delivery of gender-sensitive outpatient medical care, women veterans with a history of military sexual assault and/or posttraumatic stress symptomology perceived that they were not receiving the same quality of care as male veterans.

## Background

Approximately 456,000 women veterans utilized United States Veterans Health Administration (VHA) services during 2015, which accounted for about 7.5% of total VHA users; that percentage is expected to grow as more women enter the Armed Forces [1, 2]. Further, women who initiate VHA care are higher utilizers of healthcare services than their male counterparts, resulting in an even greater proportion of healthcare visits dedicated to the treatment of women veterans than their absolute numbers would suggest. [3]. Yet, numerous studies demonstrate room for improvements in the care of women veterans. For example, many women have accessed VHA services but later discontinued their care [4, 5]. Those who discontinued care felt unwelcome; they also perceived VHA providers to be under skilled at treating women and relatively insensitive to female veterans’ concerns [4]. Among women who used VHA mental health services, fewer than 50% believed that their mental health treatment needs were well met, and a number of women specific issues (e.g., limited or no access to women-only mental health treatment settings) contributed to that perception [6]. Further, women veterans utilizing VHA services have required more appointments (both within and outside VHA) than men in order to have their health care needs met [7].

In 2010, to improve women veterans’ access and care satisfaction, VHA mandated gender-sensitive primary care for women that included separate comprehensive women’s primary care clinics or a separate provider/team for women veterans within mixed-gender primary care clinics, co-location of mental health services with primary care, access to female chaperones for women-specific exams, and efficient access to gynecology care (either onsite or through referral) [8]. Data collected prior to and following the mandate demonstrate that disparities in women veterans’ care have been reduced [9]. However, variability in the implementation of the policy may have resulted in uneven access to needed services and satisfaction in care across facilities [10]. Furthermore, given that the policy largely focused on primary care, unmet needs may remain elsewhere, e.g., in mental health. Thus, it is important to understand the unique needs and experiences of women veterans as improvement efforts are implemented in order to ensure they have access to high-quality, patient-centered care.

One group at particularly high risk of continuing to experience disparities in care and low levels of satisfaction are women veterans with a history of sexual trauma or PTSD. PTSD is the most common condition for which women veterans receive service-connected disability, and about one quarter of women who use VHA outpatient services have a history of military sexual assault (MSA) [11, 12]. Midlife women veterans (those aged 45–54) are part of the largest age cohort of women veterans seeking care from VHA, and they are particularly likely to have experienced trauma during their military service [1, 13]. In a national study of women veterans, midlife women were significantly more likely to report experiencing MSA, sexual harassment, and physical victimization during their service than women veterans in other age groups [13]. Both a history of sexual assault and the presence of PTSD, which are prevalent in this group, are associated with increased health service utilization but decreased satisfaction with those services [14, 15]. Further, VHA care providers have reported unique barriers to delivering comprehensive care to women veterans with a history of MSA and have indicated that a history of MSA can result in delayed care [16]. As such, it is important to explore the unique experiences of this group of veterans as policies to improve the experience of all women veterans are implemented. Qualitative methods are particularly useful for facilitating a deep and nuanced understanding of complex interactions, such as healthcare experiences, which can be used to develop or refine effective policies and interventions. Thus, this study’s objective was to obtain a rich understanding of gender-specific challenges and successes encountered by midlife (e.g. Vietnam and post-Vietnam era) women veterans with PTSD and/or a history of MSA in using VHA services 1-to-2 years after the issuance of the mandate for gender-sensitive primary care services.

## Methods

This manuscript presents secondary data analyses from a larger mixed method study of a national sample of veterans who had previously applied for PTSD-related disability benefits. In this manuscript, we discuss results from women veterans who participated in the study’s qualitative portion. Specifically, we present data regarding women veterans’ experiences seeking VHA care that emerged during in-depth, semi-structured interviews that were conducted to explore veterans’ perceptions of factors that impacted the course of their PTSD symptoms and functioning. The institutional review board at the Minneapolis VA Medical Center approved the study procedures, including a waiver of written informed consent.

Participants were members of an established, nationally representative cohort of veterans who first applied for VA PTSD disability benefits between 1994 and 1998 [17, 18]. Veterans were eligible to participate in the qualitative portion of the study (semi-structured interviews) if they returned a questionnaire fielded as part of a third-wave of data collection (surveys administered in 2010–2011) and 1) indicated their willingness to participate in an in-depth qualitative interview, 2) had no change in their VA PTSD disability benefits since the first-wave survey of this cohort (1998–2000), and 3) demonstrated a clinically meaningful improvement or worsening in their PTSD symptoms and their work, role, and social functioning since the second-wave survey of the cohort (2004–2006) as the primary goal of the interviews was to explore factors associated with improvement and worsening. Of the 1014 women veterans who returned the questionnaire, 260 (25.6%) met these criteria; 48 were randomly selected to participate and 37 completed interviews. Two declined once contacted for the interviews and 9 were no longer reachable.

Interviews were conducted via telephone by professional health survey research interviewers who employed a semi-structured interview guide. Interviews lasted approximately 60 to 90 min, were audio-recorded, and were conducted between August, 2011 and December, 2012. The goal of the interviews was to ascertain whether veterans thought they were doing better or worse as compared to the time of the second-wave of the survey (2004–2006) and explore what they perceived as the most important determinants of their current well-being. Participants were asked to discuss “some of the biggest things that [had] affected” their well-being over the past several years. Following veterans’ responses to this open-ended question, interviewers largely followed the flow of a topical conversation and veterans’ narratives. In cases where additional prompts were needed to obtain a rich, thick narrative, interviewers used the interview guide to probe specific pre-specified domains (e.g., friends and family, treatment, spirituality). While the interview guide included treatment experiences as a probe, gender-specific VHA experiences were not included as a topic; instead, narratives related to that domain arose spontaneously. Veterans received $50 for completing the interview. Demographics and MSA history were collected from the surveys.

All audio-recordings were transcribed verbatim. Data were managed using NVivo versions 8 and 9 (QST International, Cambridge, MA) and were analyzed using a modified grounded-theory approach [19]. Following bottom-up, systematic coding strategies, two investigators (M.M and E.H.) sorted text segments into categories and applied pattern and thematic codes and sub-codes derived from first impressions, common phrases, and common ideas that emerged from the data. Both analysts read and coded all transcripts and met periodically to collaboratively develop and refine codes, and to condense codes into higher-order abstract concepts (e.g., themes and domains). At several points throughout the coding process, the two primary analysts met with all study investigators to refine codes and themes and examine alternative ways of looking at the data. One of the domains that emerged from this process was gender-specific VHA experiences. For the current manuscript, M.M., E.H., and the lead author (S.K-F.) further refined the themes within that domain and used constant comparison to explore similarities and differences in narratives between female veterans with and without a history of MSA. The resulting themes within the domain of gender-specific VHA experiences and representative quotes are presented.

## Results

The 37 women who completed interviews were predominately white and college educated; most reported a history of MSA (see Table 1). Of the 37 women, 30 commented on gender-specific experiences seeking VHA care (73.3% of whom reported a history of MSA). Four themes related to gender-specific VHA experiences at the time of the interviews were identified: (1) the VHA healthcare system fell short of meeting women veterans’ needs, (2) the VHA environment was unwelcoming to women, (3) improvements perceived following the implementation of women-specific clinics, and (4) suggestions for continued improvement. We describe these themes below.

**Table 1:** Characteristics of Women Veterans who Completed Semi-Structured Interviews

Characteristic	Participants (*N* = 37)
Age, mean years (SD)	54.7 (9.3)
College experience, %	91.9
Married, %	43.2
Working for pay, %	24.3
Service Era	
Vietnam, *%*	45.9
Post-Vietnam^b^, *%*	54.1
Service connected, %	89.2
Service connected for PTSD^a^, *%*	54.1
Self-reported history of Military Sexual Assault	64.9

^a^ *PTSD* Posttraumatic Stress Disorder, ^b^Includes veterans from the 1975–1998 peacetime and Gulf War I eras

### Many VHA services fell short of meeting women veterans’ needs

This theme is well-summarized by the following quotation from a 57-year-old Vietnam veteran: “I wish that women would be treated just like the men are treated.” Many women veterans expressed frustration that they didn’t have access to services that were seen as readily available to male veterans. Inpatient care for physical health services was identified as one area for which women had less timely access to care. Due to the need for either a private room or a female roommate, female veterans reported long waits for non-emergency VHA inpatient stays.

“There’s the chronic problem of the female bed… Not only did I have to wait my turn based on need, but I also had to wait until there was a female bed. So it was a long wait.” – 64-year-old Vietnam veteran.

When there was a need for emergent medical inpatient care, the lack of female beds often necessitated that women be placed in a non-VHA, community hospital. Participants stated that such community placements yielded increased paperwork and administrative burdens.

“If they use this community, immediately we get thrown into all kinds of paperwork we have to initiate the first day we’re sick; placing phone calls, getting paperwork together so the civilian doctors can be paid, trying to get them to understand that they will get paid but it has to come through the federal government and you have three days to initiate the paperwork. Now if you’re really sick, what are you going to do?” – 64-year-old Vietnam veteran.

Participants also expressed concerns about the accommodations available to women needing inpatient mental health care. Veterans stated that necessary resources (e.g., showers with hot water) were not readily available to women. Of particular concern to women with a history of MSA was sharing inpatient wards with men. One woman with a history of MSA recalled feeling unsafe during her inpatient mental health stay:

“I said ‘Do you mind if I lock my room, because I’d rather lock my door?’ And she [staff member] said, ‘No problem’…And she got in trouble for showing me how to lock my door. And I told them, ‘No, I asked for it. I don’t want my doors unlocked.’ They told me I can’t lock my door… [They should] treat some of the people that go in there, that [have] MST, with a bit more dignity.” – 58-year-old Vietnam veteran.

Women veterans with a variety of trauma types also perceived unequal access to the full range of mental health services that are available to men. The type of services most often found to be lacking were group therapy and support groups for women. Some participants reported that groups were limited to male veterans; others noted that while groups were open to women veterans, they felt uncomfortable participating in groups that were remained predominately male. Women-only groups were not available at many veterans’ clinics.

“The VA takes better care of men. They have a different program for men. They have a better support group for men than they do for women… No, I haven’t even found a support group for women.”- 50-year-old post-Vietnam era veteran.

“There’s a support group for, you know, women who’ve, you know, suffered PTSD due to rape in the Navy, and the support group is like 3 hours away. It’s like, well that’s helpful [*sarcasm*].” - 44-year-old post-Vietnam era veteran.

Finally, participants stated that they wished that they had better access to female doctors and to doctors with more experience treating women, particularly outside of women-specific primary care clinics. As one 63-year-old post-Vietnam era woman stated, “I’ve been seeing primarily male doctors who treat primarily just male patients.” The veterans felt as though this negatively impacted their care because they were not as comfortable sharing their concerns with male doctors (particularly women with a history of MSA), and the doctors weren’t as knowledgeable about gender-specific concerns or presentations.

### VHA’s predominately male environment was unwelcoming to women

Many participants expressed discomfort with being one of only a few female patients in VHA facilities. Women veterans with and without a history of MSA voiced general discomfort and mistrust associated with being a minority in the healthcare system. As one 73-year-old Vietnam era woman without a history of MSA stated, “I felt very out of place because I was female and everybody else in the place was male.” For women with MSA history, these feelings seemed stronger yet; the overwhelmingly male environment of the VHA often raised fear and distress. For some, the environment triggered trauma-related feelings and memories.

“It’s the overwhelming presence of male veterans. I don’t know them. It doesn’t matter. It triggers me.” – 52-year-old post-Vietnam veteran.

Participants with a history of MSA also noted that male veterans engaged in behaviors that increased their distress.

“At the VA, one guy walks by and [says] ‘Ooh babe, you’re looking good today.’ I got so mad, how dare you talk to me like that! But I didn’t say it out loud. [I] just kinda run because I’m scared to say it out loud! I don’t really go there by myself. My husband is usually always there, or a family member or a friend (*sniffling*)…” – 49-year-old post-Vietnam veteran.

This perception led to women feeling negatively about seeking care at VHA medical centers and, for some, to avoid VHA care altogether.

### Women veterans’ care had recently improved for some

Some participants commented on positive changes that they had recently observed. Participants reported that access to gynecological care and other -specific primary care services had improved within VHA, linking this to the implementation of primary care clinics specifically for women veterans. Women appreciated these changes when they were implemented.

“They started a women’s clinic just for women at my VA and that’s a wonderful thing. We don’t have to sit in the waiting room with all the men. It’s much better, [we] have our own little area.” – 42-year-old post-Vietnam era veteran.

Some participants had been offered personalized solutions by VHA staff that improved their experiences. For example, one 52-year-old post-Vietnam era woman with a history of MSA had trouble waiting in lines with male veterans. She described a recent individualized solution implemented at her VHA saying, “I’ll tell you what else helps, the travel office put it so I don’t have to wait in line [with men] because I get anxiety attacks when I have to do that.” Participants believed that these improvements would lead to better experiences at VHA facilities for women veterans from the Iraq and Afghanistan war eras.

### Suggestions for continued improvement

Several women offered recommendations for improving the experience of women seeking care at VHA facilities. Many of the suggestions mentioned by participants were included in the 2010 policy; however, it was clear that the improvements had not been implemented within some participants’ clinics (possibly due to the only 1-to-2-years lapse between the policy mandate and the interviews). For example, women veterans requested either having women-only waiting areas or allowing women to wait in an unused room (e.g., an exam room) prior to primary care appointments.

“I want to have a specific place I can go while I’m waiting for my general medical… that doesn’t involve that [male] population. It makes things a lot more comfortable to want to come in and utilize the VA.” 52-year-old post-Vietnam veteran.

Others recommended that gender-specific improvements be extended beyond primary clinics, such as separate waiting areas for women in mental health and specialty care clinics.

“I think I would feel much more comfortable if I was walking into a mental health clinic that was women only.” – 43-year-old post-Vietnam era veteran.

Women with and without a history of MSA requested more gender-specific programming for PTSD and related mental health concerns for women. Participants were particularly interested in more women-only groups; as one 43-year-old post-Vietnam era woman stated, “definitely female groups.” Finally, participants suggested making VHA facilities more child-friendly for returning veterans (e.g., changing tables in bathrooms).

## Discussion

Although some participants noted reasons for hope and improvement, at the time of the interviews many of the women in this sample had had recent negative experiences with the VHA that were primarily driven by a lack of gender-specific treatment options and resources. The deeply personal experiences shared by these women veterans mirrored extant quantitative literature pertaining to gender gaps in VHA care. For example, a survey of male and female veterans found that women had lower levels of satisfaction than men in a number of domains of inpatient care, including several that map onto our findings (e.g., access and physical comfort) [20]. Further, a substantial minority of surveyed female VHA mental health service users were unable to access women-only group or treatment settings as often as they wished, and a systematic review examining women veterans’ health reported that a lack of gender-specific mental health services was a barrier to women seeking VHA care [6, 21]. However, in interpreting these findings it is important to remember that the data reflect a time of transition in the delivery of women veterans’ care; it is possible that full implementation of the mandated changes took longer than the 1-to-2 years that had passed at the time of the interviews. Access to gender-specific services could have improved steadily in the time after these interviews took place. While this study is unable to directly examine this possibility, available data suggest that the concerns presented above are likely to persist. Implementation of at least one aspect of the mandate was well underway at the time of the interviews; by the time we completed our last interview, 100% of VHA healthcare systems had at least one designated women’s health provider and 63% of women primary care patients had had at least one appointment with a designated women’s health provider [22]. Yet, data collected from women veterans since 2012 suggest access to gender-specific services remains a concern. For example, a recent mixed-method study of women veterans who were past-year primary care users reported women veterans’ continue to desire gender-specific mental health programming [23].

While the women in our sample were very interested in women-only services, VHA administrators and providers have worried that segregating women veterans may lead to further stigmatization, perhaps through making them less visible and contributing to a perception of their otherness [24]. As such, any modification that serves to make women veterans less visible should include an evaluation of unintended consequences to ensure that it doesn’t contribute to the marginalization of women veterans. Inadequate resources also sometimes restrict VHA facilities’ ability to provide women-only services. Some VHA administrators and providers have had problems sustaining women’s groups due to low levels of veteran participation or a lack of qualified providers to lead groups [24]. VHA medical centers, particularly those in rural areas, might consider interactive video telemedicine as a way to congregate critical masses of women veterans with a specialized provider. Several recent studies have demonstrated the safety and efficacy of mental health treatments delivered via telemedicine [25–27]. Embedding mental health providers into women’s clinics might also effectively deliver gender-sensitive mental health treatments [24]. However, current guidelines for VHA’s primary care/mental health integration clinics recommend that veterans with chronic PTSD be referred to specialty mental health. Furthermore, the suggested number and length of primary care/mental health integration sessions does not allow for the delivery of evidence-based psychotherapies for PTSD [28]. Revising these policies and allowing increased access to evidence-based PTSD treatments within primary care may better meet the needs of this important group of veterans.

As minorities in VHA facilities, women veterans expressed attitudes similar to those that have been voiced by others seeking healthcare in a setting in which they are a minority, such as a lack of trust in providers [29]. The tenor of negative reactions differed among the women with a history of MSA. Women with a sexual assault history were particularly concerned about their safety in a predominantly male environment and discussed being “triggered” either by the presence of large numbers of men or by the inappropriate actions of individual men. In a recent study of regular primary care or women’s clinic users, one-quarter of women veterans reported unwanted interactions with male veterans while seeking care at their local VA facility [30]. Most frequently these included cat-calling, flirting, being told women don’t belong at the VA, and being propositioned for sex [30]. Many veterans—but especially those with an MSA history--would find such interactions offensive and even threatening. Among our participants with a history of MSA, being surrounded by men often served as a reminder of their trauma. Unwanted interactions with some male veterans increased their anxiety and sometimes prompted them to avoid or reduce VHA health care seeking.

Recently, a panel of experts in women’s health issues argued that VHA must ensure women veterans’ privacy, safety, dignity, and security [31]. While creating cultural change at an organization level is complex, the VHA has launched a national culture change initiative to be implemented locally by Women Veterans Program Managers [31]. Its impact is currently unknown. Vogt and colleagues have also developed and tested an educational intervention designed to improve the gender awareness of healthcare providers; results showed the intervention successfully increased gender-specific sensitivity and knowledge [32]. However, interventions aimed at reducing the frequency with which male veterans direct unwanted sexual comments toward women veterans may still need to be developed and evaluated. In addition to organizational cultural change, environmental modifications can be made to improve women veterans’ experience. The expert panel also recommended restructuring building entries and public spaces in order to ensure the comfort and safety of women veterans [31]. For example, one panelist discussed how the arrangement of waiting room chairs made women feel on display when entering one medical center; a relatively straightforward solution such as re-arranging the chairs may increase women’s comfort [31]. Facilities could also be redesigned such that women-specific clinics are located close to facility entrances or are accessible by an external door. However, as mentioned above, unintended consequences of such modifications should be evaluated.

It is possible that even with cultural change and environmental modification, some veterans, particularly those with a history of MSA, may never feel comfortable accessing VHA services in a predominately male environment. In these cases, home-based telemedicine options could be explored. Policy makers could also consider prioritization of these veterans for access to care outside of VHA through fee-basis or Veterans Choice Act community providers. Finally, personalized solutions such as letting women veterans wait in exam or group rooms, providing escorts in public areas of the hospital, or changing clinic-level processes to reduce the amount of time women have to spend in public areas of the hospital (e.g., drawing blood in clinic) may improve women veterans’ experiences and can be enacted by an individual provider or clinic. In the service of improved patient-centeredness, providers and staff should be encouraged to find personalized solutions to improve women veterans care.

Finally, some participants noted improved access to gender-specific primary care serves (e.g., gynecological care) that resulted following the implementation of women’s clinics. This finding is encouraging, both because it reflects an improvement in the care of women veterans and because it demonstrates that prior policies designed to improve women veterans’ treatment yielded the intended effect for at least some participants. Our findings suggest that future policies and improvement efforts should focus on ensuring effective implementation across all primary care settings and extending the policies to inpatient, mental health, and possibly other specialty care settings. This finding is in line with the recommendations of the expert panel described above, who identified increasing the sex awareness of everyone involved in the care of women veterans as one of the top priorities for delivering gender-sensitive care [31].

To the best of our knowledge, this is the first qualitative study specifically examining the VHA healthcare experiences of women veterans with a self-reported history of service-related trauma. Although the number of participants was small, this was a large study by qualitative standards. While women with PTSD and history of MSA are a large and important consumer subset for VHA, the women in our sample represent only the subset of these veterans who have filed PTSD disability claims. This may represent a more severe subset of women veterans with service-related trauma, as prior research suggests that disability applicants with mental disorders may have higher levels of symptomology than others with similar mental disorder who never apply [33, 34]. This study was also limited because it did not include veterans from Operations Iraqi Freedom/Enduring Freedom/or New Dawn. Our sample noted improvements in VHA services in recent years and expressed hope that newer veterans would have better experiences than they had. As this manuscript presents secondary data analyses, participants were not directly asked about gender-specific VHA experiences; their comments were unsolicited and emerged during discussions of the recent trajectories of their PTSD symptoms. It cannot be assumed that those who did not discuss it spontaneously lacked opinions or relevant experience; the themes that emerged may have been different had all women been systematically asked about gender-specific VHA experiences. We also did not specifically ask these women to compare their VHA care to non-VHA care. These women’s non-VHA care experiences might have been as negative, or even more negative, than what they reported for the VHA. More direct research with women veterans should be done to assess their impressions of VHA and non-VHA care. The present findings might serve as a useful starting point for this goal.

## Conclusions

The study demonstrates that while gains had been made in the provision of gender-specific outpatient medical care, at the time of the interviews many women veterans with a history of MSA and post-traumatic stress symptomology continued to feel uncomfortable and unwelcome in VHA facilities and perceived that they were not receiving the same quality of care as male veterans. Interventions at the system, clinic, and individual provider-level may improve the experiences of women veterans seeking VHA healthcare.

## Abbreviations

MSA: Military sexual assault
PTSD: Posttraumatic stress disorder
VHA: Veterans Health Administration
